# Atrioventricular block during exercise treadmill testing - What is the mechanism?

**DOI:** 10.1016/j.ipej.2022.12.004

**Published:** 2022-12-28

**Authors:** Sakthivel Ramasamy, Vickram Vignesh Rangaswamy, Manoj Sivaramakrishnan, Raja J. Selvaraj

**Affiliations:** a Kauvery Hospital, Alwarpet, Chennai, India; b Sri Ramakrishna Hospital, Coimbatore, India; c Jawaharlal Institute of Postgraduate Medical Education and Research, Puducherry, India

A 45-year-old female underwent treadmill testing (TMT) for evaluation of atypical chest pain, following standard Bruce protocol. Her baseline electrocardiogram (ECG) and echocardiogram were normal. Stage 1 and stage 2 of TMT progressed normally with a peak heart rate of 126/min (Fig. 1). During stage 3, ECG monitor displayed a drop-in heart rate to 57/min with 2:1 atrioventricular (AV) block (Fig. 2), however she remained asymptomatic. Further testing was terminated. Patient remained asymptomatic throughout the study period. What is the mechanism of the AV block during the TMT?

**Fig. 1:**
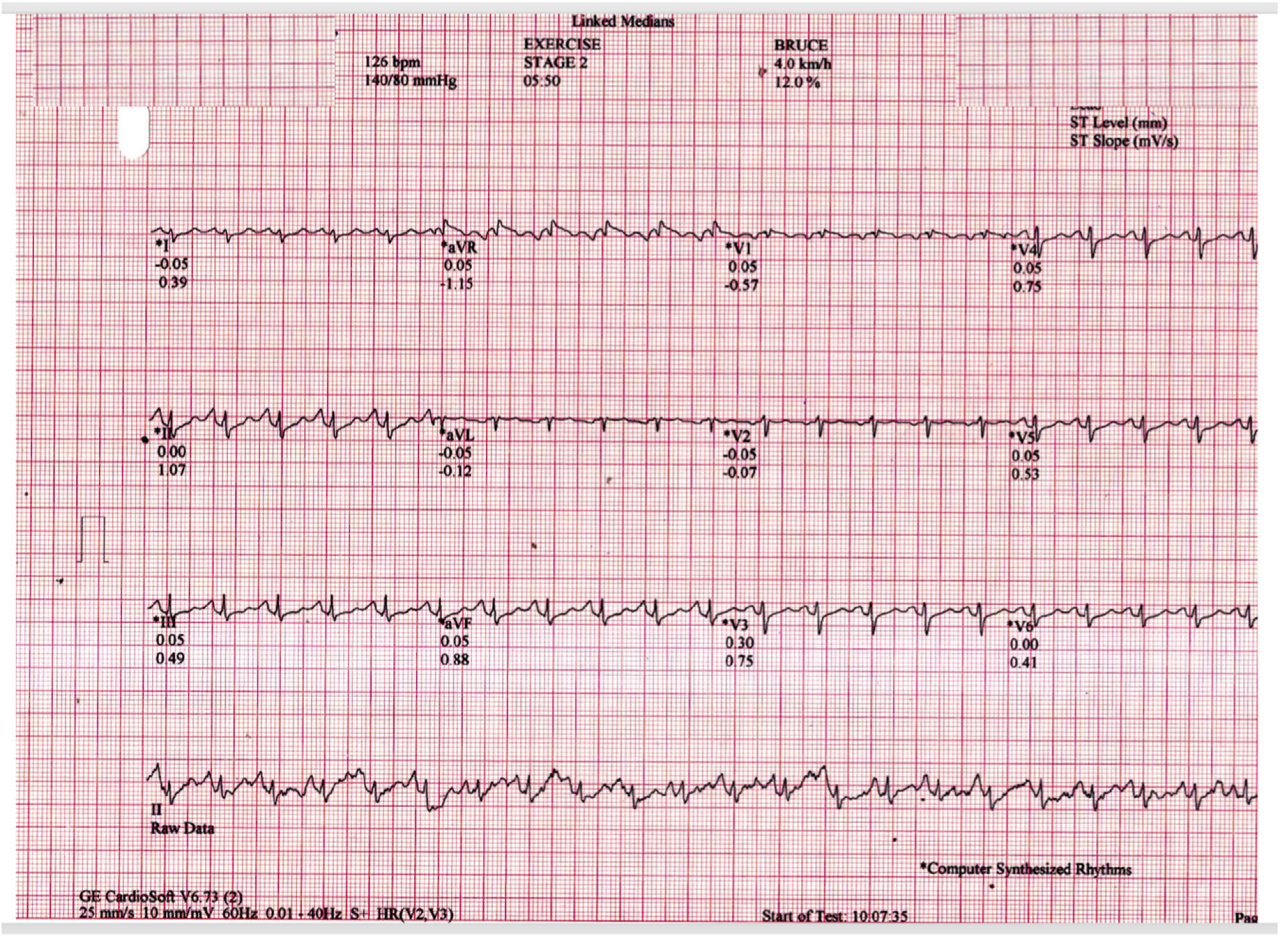
Electrocardiogram at stage 2 of exercise treadmill test.

**Fig. 2:**
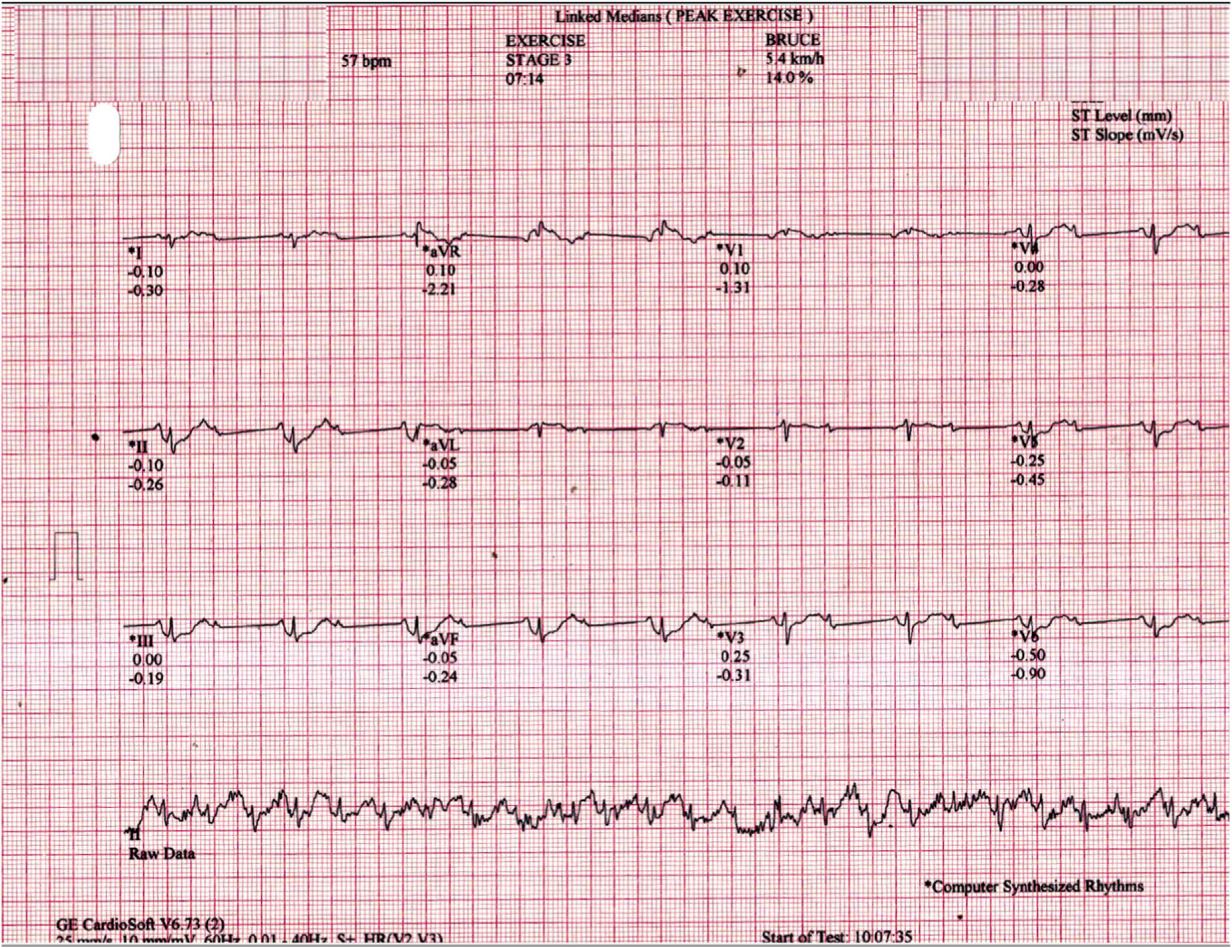
Electrocardiogram at stage 3 of exercise treadmill test.

## Answer

**Fig. 3:**
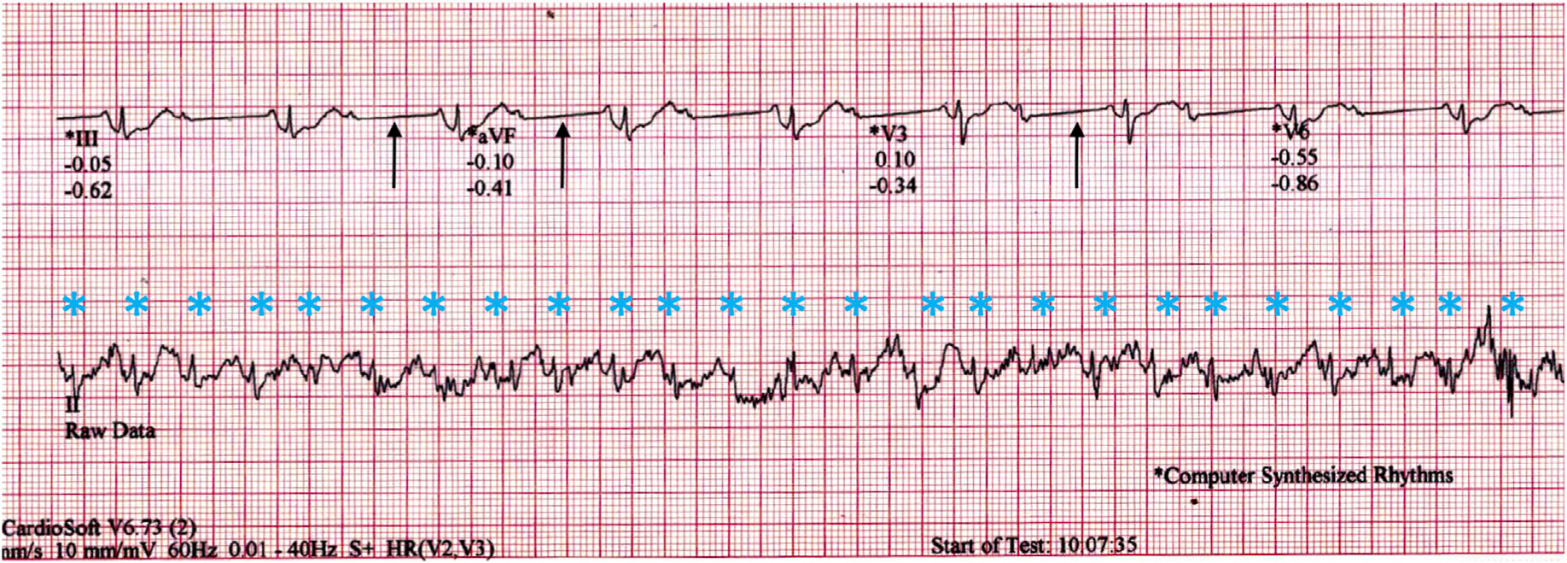
Electrocardiogram with linked median ECG (upper channels) and raw ECG (bottom channel) recorded during stage 3. (Asterisks denote QRS complexes. Arrows point to the “artificial zero” of the linking segments.).

The 12-lead linked median ECG shows heart rate of 57/min with 2:1 AV block (Fig. 2). However, concurrent lead II raw ECG tracing shows discernible P-QRS at the rate of 150/min with superimposed high frequency noise probably due to muscular activity along with baseline drift (asterisks in Fig. 3). The raw ECG represents the true cardiac activity of the patient and the occurrence of AV block in the linked median ECG is due to abnormal signal processing of the TMT machine algorithm.

Raw ECG is usually replete with background noise due to the body movement. The computer uses the signal averaging process to reconstruct these signals from the source data and display them as interpretable tracings without loss of critical information. Linked median is one of the popular methods used in the signal averaging process in TMT [1]. It works by identifying consecutive R waves amidst the background noise and the cycle length (CL) period (i.e., heart rate) is calculated. The algorithm sees the R wave as a fiducial median point with equal halves of calculated CL (i.e.CL/2) spreads back and forth as a time window and this constitutes an averaged interval. For display, the machine “links” such consecutive averaged intervals with a brief period of electrical silence (zero line) without affecting the cycle length.

**Fig. 4:**
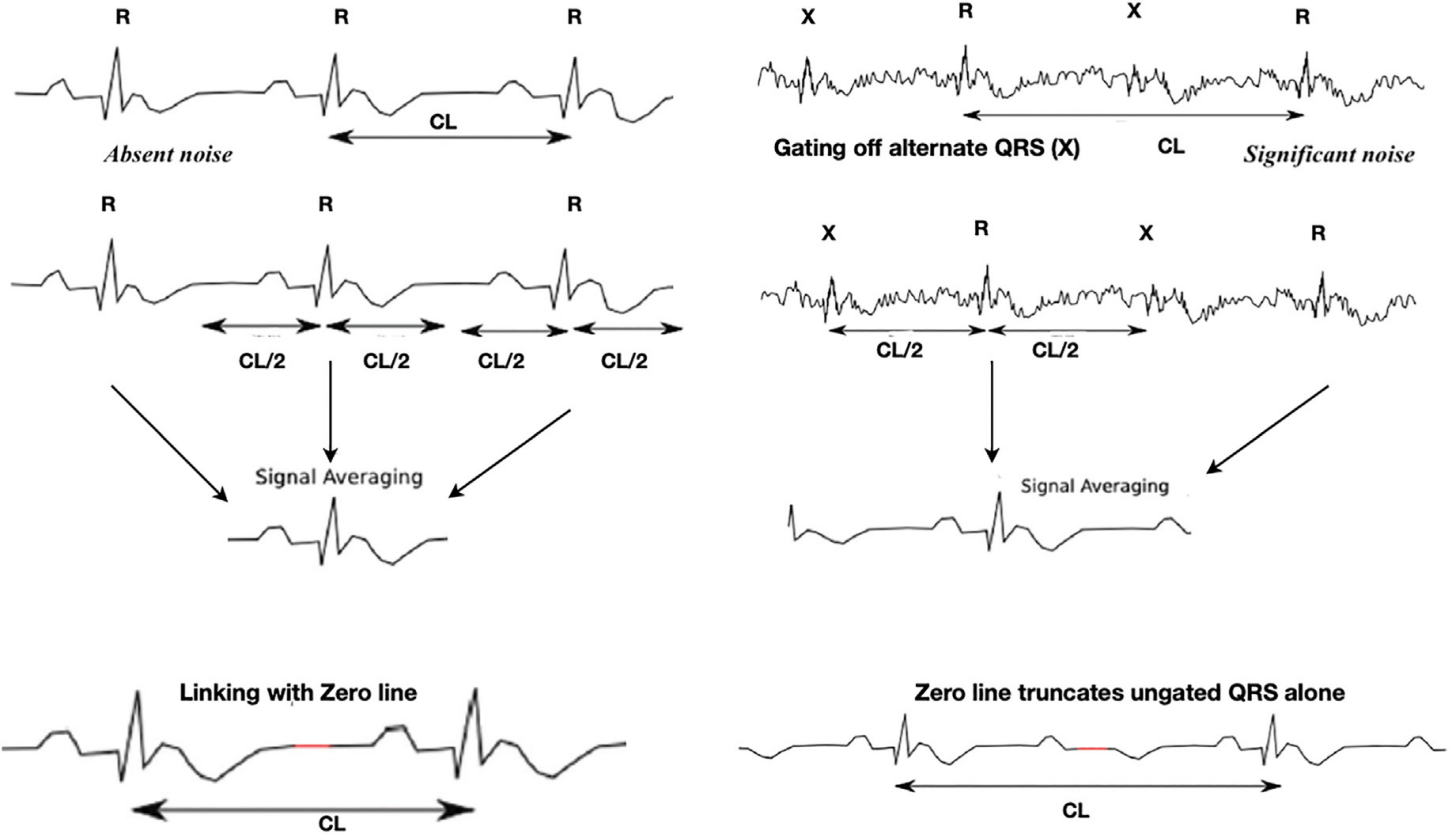
“Linked medians” reconstruction and source of artifact.

In the presence of noise, the algorithm fails to identify the alternate R waves and the CL is spuriously doubled. The resultant longer averaged segment with R wave at the centre is linked with the similar consecutive segments with long zero line. In this process, the long zero line truncates the non gated alternate QRS but preserves the P and T waves, thus giving the appearance of 2:1 AV block (Fig. 4). Sinus cycle length (PP interval) is artificially lengthened here between blocked and conducted P waves by linking with this easily appreciable long zero line [2]. (arrows in Fig. 3).

Hence it is important to compare the averaged signals with raw data in TMT testing whenever there is any abnormality [2,3]. Knowledge of these machine algorithms will help in better understanding of the artifacts and troubleshoot discordant findings.

## Declaration of competing interest

None.
